# Broadening horizons: Pathogenesis and therapeutics of renal ciliopathies

**DOI:** 10.1002/ccs3.70102

**Published:** 2026-08-12

**Authors:** Qiaowei Zhang, Shuwen Xue, Zhi Gao, Panlai Shi, Li Wang, Qi Feng, Xiangdong Kong, Xiaofan Zhu

**Affiliations:** ^1^ Department of Obstetrics and Gynecology Genetics and Prenatal Diagnosis Center The First Affiliated Hospital of Zhengzhou University Zhengzhou China; ^2^ Department of Nephrology The First Affiliated Hospital of Zhengzhou University Zhengzhou China; ^3^ Research Institute of Nephrology Zhengzhou University The First Affiliated Hospital of Zhengzhou University Zhengzhou China; ^4^ Henan Province Research Center for Kidney Disease The First Affiliated Hospital of Zhengzhou University Zhengzhou China

**Keywords:** molecular mechanisms, prenatal diagnosis, primary cilia, renal ciliopathies, signaling pathways

## Abstract

Renal ciliopathies encompass a spectrum of genetic disorders arising from structural or functional impairments of primary cilia, specialized organelles critical for mechanosensation and signal transduction within renal epithelial cells. These disorders are characterized by cystogenesis, driven by dysregulated ciliary signaling, leading to uncontrolled epithelial proliferation, aberrant growth, and loss of cellular polarity. The clinical trajectory evolves from initial cyst formation to advanced tubulointerstitial fibrosis and progressive renal failure. This progression is governed by pathogenic variants in genes encoding ciliary proteins. While advancements in genetic testing have established prenatal diagnosis as a pivotal tool for early identification, definitive diagnosis and therapeutic intervention remain challenging. These difficulties stem from several factors: incomplete understanding of the molecular mechanisms underlying cyst formation and fibrosis; limitations in prenatal diagnostic accuracy owing to phenotypic overlap and incomplete penetrance; and the marked genetic heterogeneity and diverse clinical trajectories of renal ciliopathies. Existing studies have primarily focused on unidirectional modulation of individual pathways, whereas the systematic integration of signaling network cascades remains largely unaddressed. This review systematically elucidates the molecular mechanisms and aberrant signaling pathways in renal ciliopathies, links genetic heterogeneity to clinical phenotypes, and lays a theoretical basis for prenatal diagnosis and novel therapies.

## Graphical abstract

This review elucidates the molecular mechanisms and aberrant signaling pathways in renal ciliopathies, links genetic heterogeneity to clinical phenotypes, and lays a theoretical basis for prenatal diagnosis and novel therapies.

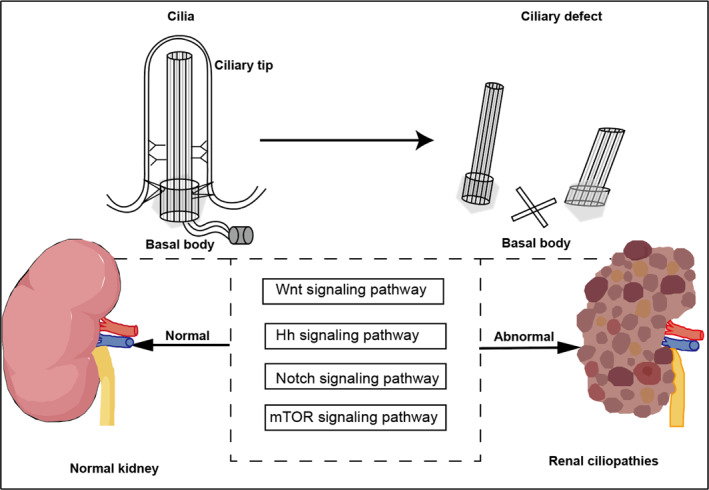

## 1 INTRODUCTION

Cilia are microscopic hair‐like organelles projecting from the apical surface of most quiescent mammalian cell types.^1^, ^2^ They serve as multifunctional sensory and signaling hubs that orchestrate critical developmental and homeostatic processes, including left‐right axis establishment, mitotic regulation, cellular differentiation programs, tissue morphogenesis, and mechanochemical signal transduction through context‐dependent molecular mechanisms.^3^

Renal ciliopathies represent a spectrum of genetically heterogeneous disorders caused by pathogenic variants affecting ciliary integrity/functions or signaling competence and are characterized by kidney dysfunction. Common renal ciliopathies mainly include autosomal dominant polycystic kidney disease (ADPKD), autosomal recessive polycystic kidney disease (ARPKD), nephronophthisis (NPHP), and Bardet–Biedl syndrome (BBS). Pathognomonic cystogenesis in these disorders is caused by dysregulated epithelial behaviors, including excessive proliferation, abnormal planar cell polarity (PCP), and basal body defects, which are mediated by impaired ciliary transduction in the Hedgehog, Wnt, and mechanosensitive signaling cascades. This cystic process, in turn, drives progressive parenchymal destruction. When coupled with maladaptive chronic inflammation, it creates a self‐perpetuating cycle of injury that inevitably advances to end‐stage renal disease (ESRD), making renal replacement therapy necessary.

With advancements in genetic testing, an expanding spectrum of genes linked to renal ciliopathies has been revealed. However, definitive prenatal diagnosis and effective treatments remain challenging. This is primarily due to an incomplete understanding of the molecular mechanisms driving cyst formation and fibrosis, compounded by limitations in prenatal diagnostic accuracy arising from phenotypic overlap, genetic heterogeneity, incomplete penetrance, and variable clinical trajectories (delayed or subtle embryonic manifestations).^4^ Existing studies have primarily focused on unidirectional modulation of individual pathways, whereas the systematic integration of signaling network cascades remains largely unaddressed.

Decoding the molecular and genetic basis underlying renal ciliopathies is essential for linking genotype to phenotypes. These new insights are key to advancing diagnostic accuracy, developing targeted therapies, and clarifying pathogenic mechanisms.

## 2 CILIA

### 2.1 Structure and function of cilia

**FIGURE 1:**
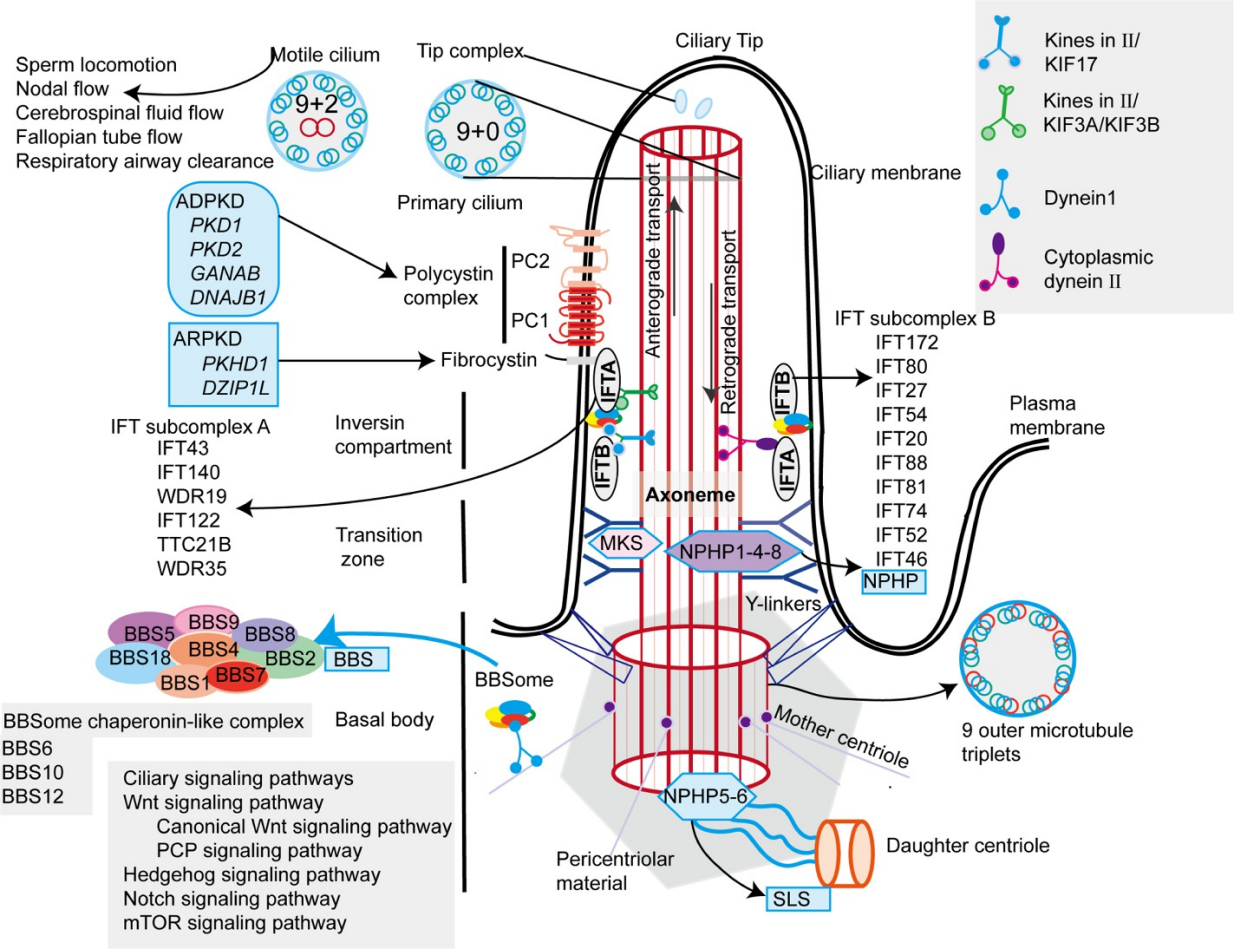
Renal ciliopathies and composition of cilium. ADPKD, autosomal dominant polycystic kidney disease; ARPKD, autosomal recessive polycystic kidney disease; BBS, Bardet–Biedl syndrome; MKS, Meckel–Gruber syndrome; NPHP, nephronophthisis; PC1, polycystin 1; PC2, polycystin 2; SLS, Senior–Loken syndrome.

Cilia can be structurally divided into subcompartments that include the basal body, axoneme, transition zone (TZ), ciliary membrane, and ciliary tip^1^ (Figure 1, Table 1). This multilevel structural integration enables cilia to function dually as molecular motors and signaling hubs. Accordingly, any compromise to ciliary structural integrity can trigger a total functional collapse.

**TABLE 1:** Ciliary structures, functions and genes linked to renal ciliopathies.

Structure	Location	Core function	Disease	Related gene
Basal body	Base of the cilium, derived from the mother centriole	Anchors the cilium to the cell surface; serves as a template for ciliary assembly; organizes microtubule arrays	JBTS	*CEP290* *OFD1*
			MKS	*CEP290* (*MKS4*) *CC2D2A* (*MKS6*)
			NPHP	*CEP290*(*NPHP6*) *SDCCAG8* (*NPHP10*)
Transition zone	Region between the basal body and axoneme; characterized by Y‐shaped connectors	Forms a ciliary–cytoplasmic barrier; functions as a “gating” region; selectively controls protein entry and exit	MKS	*MKS1* *TMEM216* *TMEM67* *TMEM107*
			NPHP	*NPHP1* *NPHP4* *CEP290* (*NPHP6*) *TMEM67* (*NPHP11*)
			JBTS	*CEP290* *TMEM67* *RPGRIP1L* *CC2D2A* *ARL13B* *INPP5E*
Axoneme	Main shaft of the cilium; composed of 9 + 0 or 9 + 2 microtubule doublets	Provides a structural scaffold; serves as the track for intraflagellar transport (IFT); mediates ciliary motility (in motile cilia)	SLS	*IQCB1* (*NPHP5*)
			LCA	*CEP290* *IQCB1*
			BBS	*BBS1* *BBS2* *ARL6* *BBS4* *BBS5* *BBS7* *TTC8* *BBS9* *BBS10* *BBS12*
Ciliary membrane	Lipid bilayer enveloping the axoneme	Enriches signaling receptors (e.g., GPCRs); mediates extracellular signal sensing; forms ciliary tip vesicles	ADPKD	*PKD1* *PKD2* *GANAB* *DNAJB1*
			ARPKD	*PKHD1* *DZIPIL*
Ciliary tip	Distal end of the axoneme	Site of ciliary assembly/disassembly; signaling processing hub; release site for ciliary ectosomes	JBTS	*KIF7*

Abbreviations: ADPKD, autosomal dominant polycystic kidney disease; ARPKD, autosomal recessive polycystic kidney disease; BBS, Bardet–Biedl syndrome; JBTS, Joubert syndrome; LCA, Leber congenital amaurosis; MKS, Meckel‐Gruber syndrome; NPHP, nephronophthisis; SLS, Senior‐Loken syndrome.

#### 2.1.1 Basal body and axoneme

Cilia are characterized primarily by two hallmark structures: the basal body and the axoneme.^5^, ^6^ The basal body is formed following mitosis, when the paired mother and daughter centrioles disengage from the spindle apparatus and migrate to a specific subplasmalemmal region where they undergo maturation.^7^ This process is centered on the mother centriole (MC), which is recruited to the site of ciliary assembly and transforms into the basal body. Ultrastructurally, the mature basal body consists of a cylindrical structure composed of nine triplet microtubules, accompanied by subdistal appendages and nine distal appendages known as transition fibers. These fibers mediate the stable anchorage of the basal body to the ciliary membrane at the proximal end of the cilium.

As a microtubule‐organizing center, the basal body plays a dual role at the base of the cilium: it acts as a structural anchor and provides a nucleation platform for axoneme assembly.^8^, ^9^, ^10^ Extending from the basal body, the axoneme forms the central cytoskeletal scaffold of the cilium, which is typically organized into nine peripheral microtubule doublets. Together, these structures support the protrusion of the cilium from the apical cell surface and enable its physiological functions.

#### 2.1.2 Transition zone

To serve as motility and/or signaling devices, cilia must be equipped with the machinery required for movement and/or signal transduction, and their composition must be distinct from that of the surrounding cell.^11^, ^12^, ^13^ However, unlike most other organelles, the ciliary membrane is continuous with the plasma membrane. To ensure compartmentalization and preserve the unique composition of the cilium, the proximal region of the axoneme encompasses a transition zone (TZ). Upon TZ formation, Y‐linkers anchor the ciliary membrane to the underlying axoneme. In conjunction with transition fibers, they establish the TZ as a “gatekeeper” and “diffusion barrier” that separates the cilia from the rest of the plasma membrane. This configuration requires active vesicle transport for molecular entry and exit,^12^ thereby forming a ciliary gate that governs the trafficking of cilium‐associated proteins.^11^, ^12^ TZ‐associated proteins, including Meckel syndrome (MKS) and NPHP module members, play crucial roles during early primary cilium formation and their dysfunction disrupts centriole migration, membrane attachment, and anchoring.^14^

#### 2.1.3 Ciliary membrane

The ciliary membrane extends from the plasma membrane and becomes distinct from the plasma membrane at the TZ.^12^ The ciliary membrane is enriched with specialized proteins and ion channels, including Ca^2+^ channels and key Hedgehog signaling pathway receptors such as PTCH1, SMO, and Gpr161.^15^ It also anchors Wnt signaling pathway receptors and the transient receptor potential (TRP) channel protein family PC1/2.^16^ Notably, mutations in the PC1/2 complex are directly linked to the pathogenesis of PKD.^17^ Consequently, despite maintaining structural continuity with the plasma membrane, the ciliary membrane forms a specialized signaling domain characterized by the following characteristics:

- Unique receptor arrays
- Compartmentalized ion flux regulators (K^+^/Ca^2+^ channels)
- Signal amplification modules (G protein‐coupled cascades)

#### 2.1.4 Ciliary tip

The ciliary tip represents a specialized subcompartment in which microtubule plus ends are localized to facilitate axonemal elongation. It also serves as the critical transition point where anterograde intraflagellar transport (IFT) (driven by kinesin) switches to retrograde transport (mediated by dynein). Furthermore, the tip harbors various signaling molecules and can dynamically alter its morphology in response to extracellular signals, underscoring its role as a signaling hub.

### 2.2 Cilia classification

Cilia exhibit two principal ultrastructural subtypes defined by axonemal microtubule doublet organization: motility‐competent cilia with a 9 + 2 configuration (containing nine peripheral doublets and a central microtubule pair) and nonmotile primary cilia, characterized by a 9 + 0 arrangement lacking central microtubules.^18^ Motile and nonmotile cilia exhibit distinct structural configurations, functional roles, and tissue distribution patterns (Figure 1).

#### 2.2.1 Motile cilia

Motile cilia exhibit structural, kinematic, and functional diversity across various cell types.^19^ Their roles span from specialized cellular locomotion to coordinated fluid propulsion.^20^, ^21^ They are prominently distributed across the cerebral ventricles, respiratory mucosa, and reproductive system,^22^ where they modulate local hydrodynamic microenvironments to support tissue homeostasis, substance transport, signaling, and asymmetric embryonic development.

The function of motile cilia arises from the precisely regulated architecture of the axoneme, which enables spatiotemporally coordinated activity of inner and outer dynein arm isoforms along the axonemal microtubules. Through Adenosinetriphosphate (ATP) hydrolysis, these dynein isoforms drive repetitive microtubule sliding, a mechanical process that depends on tightly regulated cycles of motor domain engagement and disengagement.^23^ This mechanism enables motile cilia to perform a spectrum of hydrodynamic functions, ranging from directional fluid propulsion to directed cellular migration.^24^ Thus, motile cilia not only act as locomotory organelles in specialized cells but also widely serve as microfluidic pumps in epithelial tissues, participating in developmental regulation and physiological homeostasis through mechanotransduction‐based signaling.^1^

#### 2.2.2 Primary cilia

Primary cilia are nonmotile, microtubule‐based structures protruding from the cell surface whose essential function is to serve as cellular “signaling antennas” or “signaling hubs”.^25^ They detect and transduce chemical, mechanical signals, and other extracellular cues, thereby orchestrating diverse cellular functions and regulating development and tissue homeostasis.^26^ Receptors and signaling molecules^24^, ^27^, ^28^ are highly enriched within primary cilia and sequestered from the rest of the plasma membrane, establishing these organelles as signaling hubs that convert extracellular information into downstream intracellular pathways and trigger appropriate physiological responses. The sensory capacity of primary cilia relies on the coordinated trafficking and precise spatiotemporal regulation of specific receptors and their associated signal transduction modules. Defects in primary ciliary signaling give rise to a group of pleiotropic disorders termed ciliopathies, which affect multiple tissues and organs throughout the body. Notably, defects in primary cilia are the main cause of renal ciliopathies.^29^

### 2.3 Intraflagellar transport

Owing to cilia’s inability to synthesize their own proteins, they rely on the IFT system, which uses “train‐like” IFT transporters to mediate vesicular protein trafficking within the cilium. Specifically, IFT is a sophisticated bidirectional trafficking system that operates within the confined space between the ciliary membrane and the ciliary axoneme. This highly regulated process ensures the precise delivery of essential components necessary for ciliary maintenance, assembly, and function.^8^

Anterograde transport, the movement of materials from the base of the cilium toward the tip, is predominantly mediated by the kinesin‐2 motor protein family.^30^, ^31^ Key members of this family, including KIF3A and KIF3B, along with their associated adapter protein KAP, form a functional unit that efficiently transports axonemal precursor components and other vital cargoes along the microtubule tracks of the cilium. This directional transport is crucial for the elongation and structural integrity of the cilium. Conversely, retrograde transport, the reverse movement of materials from the cilium tip back to the cell body, is executed by cytoplasmic dynein‐2 (Dyn2). This motor protein complex, composed of subunits (DYNC2H1/DYNC2LI1/D2LIC/WDR34/LC8), facilitates the recycling of components that have been sequestered at the ciliary tip or replaced during ciliary turnover. This process is essential for maintaining the cellular homeostasis of ciliary components and preventing their accumulation within the cilium. IFT particles, the structural and functional units of the IFT system, are organized into two distinct complexes: complex A and complex B. Complex A encompasses six ciliary‐specific components, whereas complex B comprises 10 such components and is instrumental in transporting outer dynein arms essential for motile cilia/flagella.

In summary, IFT complex B collaborates with kinesin‐2 to drive anterograde transport, whereas IFT complex A works in concert with cytoplasmic dynein to facilitate retrograde transport.^8^ This intricate interplay between IFT complexes and motor proteins ensures the dynamic and precise regulation of ciliary composition and function, highlighting the critical role of IFT in the biology of cilia.^32^

### 2.4 Ciliogenesis

The dynamic processes of ciliary assembly and disassembly are tightly coupled with the progression of the cell cycle.^33^, ^34^, ^35^ Ciliary biogenesis is characteristically synchronized with the G0/G1 phase transition. In contrast, ciliary disassembly is triggered upon S phase entry, serving as a prerequisite for the cell’s commitment to the mitotic cycle. This tight spatiotemporal coupling highlights the profound interplay between ciliary formation and cell division. Disruptions in cell division can impair the normal process of ciliary development, resulting in the absence of cilia in cells, a hallmark feature observed in tumors. Conversely, cells with defective cilia may undergo abnormal proliferation, with PKD serving as a prime example of a condition linked to ciliary dysfunction.

The functional versatility of primary cilia originates from the meticulous regulation of their dynamic assembly‐disassembly cycle. This homeostatic equilibrium underlies the spatiotemporal precision of the localization and functional activation of proteins associated with cilia. This dynamic cycle drives ciliogenesis, orchestrating the intricate steps required for the formation and maintenance of functional primary cilia in diverse cell types. In the absence of mitogenic factors or differentiation signals, cells exit the mitotic phase and enter the G0 phase. Ciliogenesis is subsequently initiated. Initially, within several minutes of mitogen deprivation, vesicles originating from the Golgi apparatus or recycling endosomes, specifically distal appendage vesicles (DAVs), accumulate at the distal appendage of the MC. This event triggers the transformation of the MC into the basal body, establishing a foundation for ciliary assembly. DAVs converge and fuse at the MC to give rise to the ciliary vesicle (CV).^36^ CV formation indicates maturation of the basal body. The distal appendage protein Cep164 plays a crucial role in preserving the integrity of this structure and facilitates its fusion with the CV by binding to the Rab8 and Rabin8.^37^, ^38^ In the subsequent phase of ciliary assembly, Cep164 recruits TTBK2 to the MC. Accurate localization of TTBK2 is essential for the removal of the key inhibitor CP110 from the MC, thereby enabling the recruitment of the IFT complex. This complex is responsible for the bidirectional transport of protein cargo along axonemal microtubules, which in turn promotes the assembly of the axoneme.^38^, ^39^, ^40^

Following CV maturation, the TZ begins to form at the distal end of the basal body and subsequently integrates into the CV, contributing to CV expansion. Subsequently, the ciliary axoneme is enveloped by the CV and extends longitudinally. The elongating axoneme is then covered by the ciliary sheath and ultimately fuses with the cell membrane. The fully mature cilium comprises axonemes consisting of microtubules and associated proteins, a ciliary membrane that is continuous with the cell membrane, and various interaxonemal matrices.

## 3 RENAL CILIOPATHIES

Renal ciliopathies are defined by renal dysfunction that arises from structural or functional impairments of the primary cilium.^41^ The spectrum of renal ciliopathies includes ADPKD, ARPKD, NPHP,^42^ BBS, and other renal ciliopathy syndromes (Table 2), all of which share the common clinical manifestation of renal cystic pathology. During ciliogenesis, the NPHP complex, the BBSome, and other ciliary cystoproteins localize to the centrosome or ciliary base body,^43^, ^44^ where they orchestrate key steps in ciliogenesis and ciliary protein trafficking. Loss‐of‐function or gain‐of‐function mutations in genes encoding these components disrupt their localization or impair their molecular functions, leading to defects in ciliogenesis. These defects manifest as impaired ciliogenesis, mislocalization of ciliary proteins, and dysfunction of associated signaling pathways. Consequently, disrupted signaling cascades induce epithelial hyperproliferation and cystogenesis through dysregulation of cell cycle progression, resistance to apoptosis, and altered epithelial–mesenchymal transition dynamics. With the progression of cystic degeneration in the renal parenchyma and the development of systemic inflammatory responses, these diseases often progress to ESRD, ultimately necessitating renal replacement therapies.

**TABLE 2:** Summary of major renal ciliopathy syndromes.

Disease	Inheritance	Incidence	Typical age onset	Renal manifestations	Extrarenal manifestations	Major genes	Animal models
ADPKD	AD	1 in 1000	Adulthood (30–40 years)	Gross bilateral kidney enlargement Numerous large, irregular parenchymal cysts (which can become painful and/or infected), and progressive decline in kidney function Cysts originate primarily from collecting duct/distal tubule Chronic inflammation Kidney failure: 50% by age 60	Hepatic cysts Valvular heart disease Cerebral aneurysms Hypertension	*PKD1* (78%) *PKD2* (15%) *GANAB* *DNAJB1*	Mice Pigs Rats Zebrafish
ARPKD	AR	1 in 20,000	Antenatal, early childhood or neonatal	Gross bilateral kidney enlargement Irregular cysts Poor corticomedullary differentiation Interstitial fibrosis Chronic inflammation Kidney failure: 50% by age 10	Congenital hepatic fibrosis Portal hypertension Caroli syndrome Pancreatic fibrosis Hypertension	*PKHD1* *DZIPIL* *TULP3*	Mice Rats Zebrafish
NPHP	AR	1 in 50,000–1 in 1,000,000	Infantile (<20%): ∼1 year Juvenile (80%): 4–6 years Adult‐onset	Bilateral corticomedullary cysts Chronic inflammation Tubular interstitial fibrosis Tubular atrophy Thickened basement membrane, polyuria, polydipsia Kidney failure (infantile: By 3 years; juvenile: ∼13 years)	Senior‐Loken syndrome Congenital hepatic fibrosis Short‐ribs Polydactyly Cone‐shaped epiphyses Cardiac defects	*NPHP1* (20%–50%) *TMEM67* *CEP290*	Mice Zebrafish
BBS	AR	1 in 140,000–1 in 160,000	Infantile Early childhood (60%): ∼2–5 years Childhood to Adolescence (>5 years)	Hydronephrosis, renal dysplasia Cystic kidneys Agenesis Horseshoe kidney Ectopic kidney Neurogenic bladder Urine concentrating defect Chronic tubulointerstitial nephropathy Pelvic dilation Ureter obstruction	Retinal dystrophy Obesity Polydactyly Intellectual disability Hypogonadism Delayed development Growth retardation Hearing loss Cardiac abnormalities	*BBS1* *BBS10* *BBS2*	Zebrafish Mice Rhesus Macaques Dog
JBTS	AR XLR (*OFD1*)	1 in 80,000–1 in 100,000	Infantile, neonatal	NPHP (nephronophthisis with tubulointerstitial fibrosis)	Molar tooth sign: hypoplasia of the cerebellar vermis, thickened cerebellar peduncles, and deepened interpeduncular fossa Hypotonia Ataxia Oculomotor apraxia Intellectual disability Liver fibrosis Polydactyly Facial dysmorphisms	*CEP290* (38%) *TMEM67* *OFD1*	Mice Zebrafish Frog
MKS	AR	1 in 13,250–1 in 140,000	Prenatal period	Large polycystic kidneys	Occipital encephalocele Severe postaxial polydactyly Anencephaly or microcephaly Congenital heart defects Pulmonary hypoplasia Male genital defects	*MKS1* *TMEM67* (12%–20%) *TMEM216* *CC2D2A* *CEP290*	Mice Zebrafish

Abbreviations: AD, autosomal dominant; ADPKD, autosomal dominant polycystic kidney disease; AR, autosomal recessive; ARPKD, autosomal recessive polycystic kidney disease; BBS, Bardet–Biedl syndrome; ESRD, end‐stage renal disease; JBTS, Joubert syndrome; MKS, Meckel‐Gruber syndrome; NPHP, nephronophthisis; PCP, planar cell polarity; XLR, X‐linked recessive.

### 3.1 ADPKD

With a prevalence of approximately 1 in 1000,^45^ ADPKD is the most common inherited kidney disease in adults. They are characterized by the progressive growth of numerous large, fluid‐filled bilateral renal cysts and typically present in adulthood (30–40 years) with gross kidney enlargement and hypertension. While most cases present in adulthood, an estimated 1%–5% exhibit an earlier‐onset form identified incidentally and associated with glomerulocystic alterations.^46^ Approximately 50% of affected individuals progress to ESRD by age 60.^47^ *PKD1* and *PKD2* ^48^ mutations account for ∼78%^49^ and 15% of cases, respectively. These mutations result in dysfunction of the PC1 and PC2 proteins, which co‐localize and interact at the primary cilium. Consequently, dysregulation of transepithelial fluid secretion, heightened proliferation of tubular epithelial cells, and disruption of cellular polarity and differentiation, all of which contribute to the cystic phenotype characteristic of ADPKD.^50^, ^51^, ^52^ PC1 is a large G protein‐coupled receptor (GPCR).^53^ PC2, a TRP channel,^54^ acts as a receptor‐coupled, nonselective calcium‐permeable channel. Although PC1 and PC2 exhibit distinct structural features, they are both essential to the disease pathogenesis. It was initially proposed that in PKD, the polycystin complex acts as a mechanoresponsive sensor for calcium signaling, reflecting a role for PC2 in sensing nodal flow for left‐right determination during embryonic development and fluid‐shear stress.^55^, ^56^, ^57^ PC1 and PC2 form an ion channel complex that is essential for maintaining intracellular Ca^2+^ homeostasis at the ciliary membrane. Their dysfunction results in reduced intracellular Ca^2+^ levels, triggering a signaling cascade characterized by increased cyclic adenosine monophosphate (cAMP) activity. Aberrant cAMP signaling, along with other dysregulated pathways such as mammalian target of rapamycin (mTOR), drives disoriented cell proliferation and ultimately promotes cyst formation. Besides, aberrant conformational alterations or subcellular trafficking of PC1 and PC2 receptors to the primary cilium can compromise structural integrity and functional efficacy, thereby disrupting intricate cellular signaling networks and compromising cell polarity regulation, ultimately leading to ciliopathies. ADPKD demonstrates significant phenotypic heterogeneity, which arises from the extensive allelic diversity at the *PKD1* and *PKD2* loci,^58^ as well as the complex interplay between immutable risk factors and acquired comorbidities.^47^

A minority of atypical ADPKD cases arise from mutations in other genes, such as *GANAB*,^59^ *DNAJB11*,^60^ *HNF1B*,^61^ *IFT140*, and *COL4A1*. They typically manifest as unilateral, asymmetric, or segmental renal cysts on imaging, often accompanied by extrarenal cysts in the liver, spleen, or pancreas, and show generally slower disease progression.

In the diagnosis and management of ADPKD, renal ultrasonography is the first‐line imaging tool, as it detects multiple renal cysts and intrarenal hyperechoic regions. Magnetic resonance imaging (MRI) is an important complementary modality for the more precise evaluation of the renal parenchyma and function in urological abnormalities.

### 3.2 ARPKD

ARPKD is a rare inherited cystic kidney disorder that occurs in approximately 1 in every 20,000 births. Compared with ADPKD, it typically manifests in neonates or childhood, with a more severe clinical course. The characteristic features of ARPKD include bilateral renal cysts, poor corticomedullary differentiation, enlarged and echogenic kidneys, and hepatic fibrosis, which can potentially lead to portal hypertension. Pathologically, progressive cyst expansion leads to renal parenchymal injury, characterized by interstitial fibrosis and tubular atrophy. These structural changes ultimately result in progressive and irreversible loss of renal function, leading to renal failure.^62^ *PKHD1* and *DZIP1L*,^63^ which encode fibrocystin and DZIP1L proteins, respectively, are the major causative genes of ARPKD. Besides, *PKHD1* ^64^ has a tissue‐specific transcriptomic landscape characterized by robust expression in renal tissue, where it encodes fibrocystin that predominantly localizes to the primary cilium, a critical component of renal epithelial cell signaling pathways.^65^, ^66^ This characteristic aligns it with other ciliary proteins associated with cystic kidney disease, commonly referred to as “cystoproteins”.^67^ During embryogenesis, fibrocystin is actively expressed and plays an indispensable role in normal branching morphogenesis of the ureteric bud, which is a critical process for embryonic kidney development.^68^ Notably, fibrocystin is present in both adult and fetal kidney tissues. Functionally, it serves as a pivotal regulator of cell proliferation, apoptosis, and polarization.^69^ When fibrocystin is mutated, its regulatory functions are disrupted, leading to cystic kidney disease. *DZIP1L* localizes to the “TF matrix” at the transition fiber‐zone interface and works synergistically with ANKRD26 to stabilize the ciliary gate component FBF1, thereby preserving selective protein import into the cilium. When *DZIP1L* is mutated, it fails to target transition fibers, resulting in matrix dissolution, ciliary gating failure, the exclusion of polycystin‐2 from the cilium, and ultimately, the initiation of renal cyst formation.^63^ Furthermore, ARPKD is associated with increased expression levels of epidermal growth factor (EGF), epidermal growth factor receptor (EGFR), and transforming growth factor‐α. Early signs of ARPKD are frequently detected prenatally during the second or third trimester through renal ultrasonography, which reveals enlarged kidneys with echogenicity and medullary hyperechogenicity.

### 3.3 NPHP

NPHP is a rare autosomal recessive ciliopathy classified as a hereditary tubulointerstitial nephropathy, with an estimated incidence ranging from 1 in 50,000 to 1 in 1,000,000 individuals. This disease is pathologically characterized by progressive tubulointerstitial fibrosis, inflammatory infiltration, and tubular atrophy. It is frequently accompanied by the development of renal cysts. The canonical nephrocystin modules consist of two distinct complexes: the NPHP1–4–8 module and the NPHP5–6 module. The NPHP1–4–8 module is localized to the ciliary TZ and is regulated by *NPHP8*,^70^ with *NPHP1* encoding an adapter protein that maintains interactions with other cystoproteins, participating in signal transduction and cell adhesion processes.^71^ In contrast, the NPHP5–6 module operates at the ciliary basal body, where it plays critical roles in ciliogenesis and maintenance of ciliary function through structural and regulatory mechanisms.^72^, ^73^ Clinically, on the basis of age of onset and underlying genetic etiology, NPHP can be categorized into three major subtypes.^74^ Infantile NPHP, accounting for fewer than 20% of cases, manifests within the first year of life and progresses to ESRD by 3 years of age, frequently accompanied by kidney enlargement. Juvenile NPHP presents between the ages of 4 and 6 years, with kidney failure typically occurring at approximately 13^75^ years of age. Adult‐onset NPHP shares similar histopathological features with the juvenile form but presents later in life. Juvenile NPHP represents the most common genetic cause of end‐stage kidney failure in children and adolescents. Diagnosis is facilitated by renal ultrasonography, which reveals a normal kidney size with increased echogenicity and impaired corticomedullary differentiation.

### 3.4 BBS

BBS is a multisystem ciliopathy with an estimated incidence of 1 in 140,000 to 1 in 160,000 live births.^76^ This syndrome can manifest with various kidney abnormalities, including horseshoe kidneys, fetal lobulation, kidney dysgenesis, and cystic kidneys. The clinical manifestations of BBS are characterized primarily by retinal degeneration and multiorgan damage, with more than 20 pathogenic genes identified, including those encoding BBS proteins (BBS1–BBS21). Some proteins interact to form the BBSome complex, which is localized to the ciliary basal body and functions as a cargo adapter, facilitating the assembly of IFT particles to mediate protein transport within cilia and thereby maintaining ciliary stability,^44^, ^77^ whereas the BBSome chaperonin‐like complex can promote BBSome assembly. Mutations in BBS proteins result in the mislocalization of key molecules critical for ciliary formation and function, disrupting downstream signaling pathways and leading to abnormal developmental patterns and cell cycle dysregulation. Among reported BBS cases, mutations in *BBS1* are the most prevalent, with *BBS1*, *BBS2*, *BBS4*, and *BBS7* interacting with renal tissue‐specific proteins.^78^ Besides, the BBSome interacts with other ciliary complexes, such as PC1/PC2 and NPHP, likely facilitating their ciliary targeting. Beyond this core machinery, BBS can also result from mutations in genes outside the BBSome or in genes that overlap with other ciliopathies (e.g., *CEP290, CEP164*), underscoring the marked genetic heterogeneity of this disorder, arising from perturbations in ciliary transport and associated regulatory networks.

### 3.5 Others

Joubert syndrome (JBTS) and MKS, which are both classified as ciliopathies arising from primary ciliary dysfunction, exhibit distinct clinical trajectories and genetic architectures.

JBTS is a renal‐retinal‐cerebellar syndrome characterized primarily by cerebellar and brainstem malformations. Neuroimaging reveals the diagnostic “molar tooth sign” on axial MRI.^79^ Clinically, affected individuals present with axial hypotonia, global developmental delays, and respiratory dysregulation, along with multisystem involvement. Renal involvement, observed in a subset of patients, typically manifests as NPHP with tubulointerstitial fibrosis. Genetically, JBTS demonstrates marked heterogeneity, predominantly following AR inheritance,^80^ although X‐linked (e.g., *OFD1*)^81^ and digenic mechanisms have been documented. Moreover, the encoded proteins share a common localization to primary cilia or centrosomes.

MKS is a lethal perinatal ciliopathy^82^ defined by the classic triad of occipital encephalocele, polycystic renal dysplasia, and polydactyly. The prognosis is uniformly dismal, with perinatal or neonatal death (median survival < 1 week) secondary to multiorgan failure. The genetic landscape of MKS is exceptionally complex. Although the repertoire of known causative genes continues to expand, nearly 40% of patients lack a definitive molecular diagnosis, pointing toward the involvement of rare or private variants.^82^ Renal pathology, which is virtually universal in MKS, manifests as severe cystic dysplasia with cortical‐medullary effacement and multifocal cysts.

## 4 KEY SIGNALING PATHWAYS INVOLVED IN CILIARY FUNCTION REGULATION

Cilia detect extracellular signals, such as growth factors, hormones, and other stimuli, through a group of specifically localized cilia‐associated receptors, including Wnt, Hedgehog, transforming growth factor β (TGFβ), and platelet‐derived growth factor receptors. These receptors transduce extracellular signals into intracellular signaling cascades, which activate locally enriched effectors. These effectors then coordinate downstream events to regulate tissue differentiation, homeostasis, cell proliferation, growth, and organ development and function. Disruptions in these signaling pathways have been implicated in ciliopathies.^28^, ^83^

### 4.1 Wnt signaling pathway

#### 4.1.1 Canonical Wnt signaling pathway

The Wnt/β‐catenin signaling pathway, whose components are localized to the primary cilium, critically regulates embryonic kidney development by orchestrating the proliferation, differentiation, and polarization of renal mesenchymal cells.^84^ This pathway governs glomerular size, nephron number, and renal morphology from embryogenesis to adulthood. In vitro, Aquaporin‐1 (AQP1) overexpression downregulates β‐catenin and cyclin D1, both of which interact with core pathway components (GSK3β; LRP6). Consistently, knockout of AQP1 enhances renal Wnt signaling activity in a PKD mouse model, increasing kidney size and promoting cyst formation.^85^ Collectively, these findings reveal that AQP1 suppresses cystogenesis by negatively regulating canonical Wnt signaling, with reduced β‐catenin and cyclin D1 as key downstream events. β‐catenin, a key effector of this signaling pathway, mediates signal transduction to promote renal tubule formation and nephron differentiation while regulating cellular proliferation.^86^ As an essential component of intercellular adhesion junctions (E‐cadherin complexes), it maintains the polarity of renal tubular epithelial cells. In the inactive state, β‐catenin predominantly localizes to the cell membrane or cytoplasm, where it undergoes phosphorylation and degradation by the destruction complex (APC/Axin/GSK3β). Upon activation of the Wnt pathway, β‐catenin escapes degradation, accumulates, and is translocated to the nucleus. Studies have demonstrated that when the canonical Wnt signaling pathway is inhibited by the exogenous Wnt antagonist Dkk‐1, there is a significant reduction in nascent glomeruli and marked suppression of ureteric bud morphogenesis, ultimately leading to renal hypoplasia.^17^, ^87^ Consistently, β‐catenin knockout mice exhibit varying degrees of urogenital system dysplasia, and the severity of these developmental defects is directly correlated with the level of β‐catenin expression.^88^

During kidney development, the Wnt signaling pathway exerts precise spatiotemporal regulation of multiple ligands. In early nephrogenesis, Wnt11 is expressed at the tips of the ureteric bud and regulates ureteric branching morphogenesis via a positive feedback loop with GDNF/Ret.^89^, ^90^ Subsequently, Wnt9b, expressed in the Wolffian duct epithelium and later in the ureteric bud stalk,^91^ acts upstream of Wnt4 and serves as the initial inducer of mesenchymal‐epithelial transition during urogenital development.^92^ Through paracrine signaling, Wnt9b induces the expression of Wnt4, Pax8, and Fgf8 in the ventral cap mesenchyme and promotes mesenchymal cell proliferation, thereby regulating the differentiation and expansion of nephron progenitor cells. Beyond this early role, Wnt9b subsequently signals through the noncanonical pathway to regulate PCP during renal tubule morphogenesis.^93^ Wnt4, which is present in the condensing mesenchyme and pretubular aggregates,^94^, ^95^ drives MET^96^ and is essential for nephron epithelialization. Notably, the formation of these Wnt4‐expressing pretubular aggregates is dependent on FGF8 signaling, as inactivation of FGF8 disrupts aggregate formation, thereby impeding the development of nephron precursors and the emergence of S‐shaped bodies.^97^, ^98^ Wnt5a signaling through Ror2 regulates intermediate mesoderm extension and ureteric‐mesenchymal interactions,^99^ and its deficiency leads to renal hypoplasia, kidney fusion, or kidney duplex. Wnt2b is expressed in perirenal mesenchymal cells and stimulates ureteric development.^100^ Wnt6 is expressed in the ureteric bud and induces tubulogenesis,^101^ and Wnt7b mediates the establishment of the corticomedullary axis.

**FIGURE 2:**
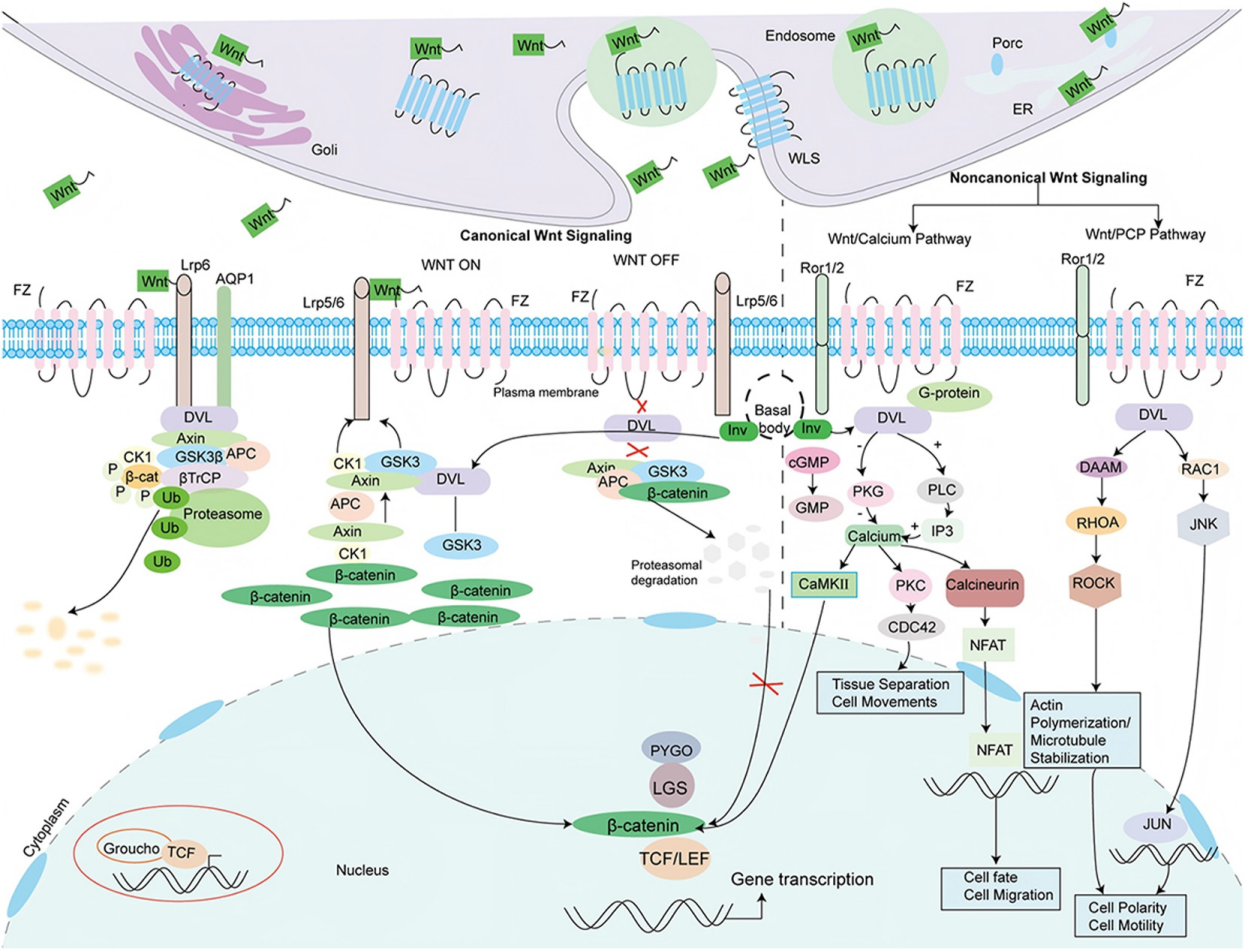
Wnt signaling pathway. Wnt signaling pathways include canonical and non‐canonical Wnt signaling pathways. In the canonical pathway, when Wnt signaling is inactive, β‐catenin is sequestered by a destruction complex composed of APC, Axin, GSK3β, and CK1, leading to ubiquitin‐mediated proteolysis. Upon Wnt binding to Frizzled and LRP5/6, a phosphorylation cascade involving Disheveled inactivates the destruction complex, allowing β‐catenin to accumulate and translocate to the nucleus, where it interacts with TCF/LEF transcription factors to regulate gene expression involved in cell proliferation, differentiation, and survival. Inversin also serves as a molecular switch between canonical and noncanonical Wnt pathways. The noncanonical pathways include the Wnt/Ca^2+^ pathway and the PCP pathway. The PCP pathway, mediated by Frizzled and Disheveled, activates small GTPases such as Rho and Rac, thereby regulating cytoskeletal rearrangement and tissue polarity. The Wnt/Ca^2+^ pathway triggers intracellular calcium release, activating calcium‐sensitive signaling molecules such as CaMKII and PKC, which influence cell fate and migration. AQP1 modulates canonical Wnt/β‐catenin signaling by stabilizing the destruction complex (APC/Axin/GSK3β/CK1), thereby promoting β‐catenin phosphorylation, ubiquitination, and proteasomal degradation. Conversely, reduced stability of the destruction complex leads to β‐catenin accumulation, nuclear translocation, and TCF/LEF‐mediated transcription. PCP, planar cell polarity; PKC, protein kinase C.

Signal transduction occurs via Wnt ligand palmitoylation by porcupine and WLS‐mediated secretion, followed by Frizzled/LRP receptor binding, cytoplasmic Dvl/GSK‐β/AXIN/APC‐mediated β‐catenin stabilization, and nuclear TCF/LEF‐dependent target gene activation.^102^ Dysregulation of this pathway is strongly associated with various congenital anomalies of the kidney and urinary tract (CAKUT). In animal models, mutations in Wnt4 lead to renal agenesis or dysgenesis. Mice harboring Wnt9b mutations exhibit nephron deficiency or cystic kidneys.^91^ Similarly, Wnt5a‐mutant mice display congenital anomalies of the kidney and urinary tract (CAKUT) phenotypes, and mutations in WLS result in cystic medullary hydronephrosis.^103^ Clinically, SERKAL syndrome, Robinow syndrome, bilateral renal agenesis or hypoplasia, and Zaki syndrome are caused by mutations in Wnt4, Wnt‐5a, homozygous Wnt9b, and WLS, respectively (Figure 2, Table 3).

**TABLE 3:** Wnt signaling components in kidney development and associated phenotypes.

Category	Component	Function in kidney development	Animal model phenotype	Human disease/syndrome
Extracellular ligands	Wnt2b	Stimulates ureteric development but does not induce tubule formation	‐	‐
	Wnt3	Affects cloaca development	Wnt3^−/−^ mice fail to develop mesoderm Wnt3 knockdown in zebrafish causes cloaca malformations	Tetra‐amelia syndrome
	Wnt4	Initiates nephron development, drives MET, regulates nephron epithelialization, and regulates smooth muscle cell fate	Wnt4^−/−^ mice die within 24 hours after birth, exhibit renal dysgenesis and undifferentiated mesenchyme	SERKAL syndrome
	Wnt5a	Regulates intermediate mesoderm extension and MM‐WD interaction, and controls collecting duct patterning via Ror2 signaling	Wnt5a^−/−^ mice display various CAKUT phenotypes Wnt5a knockdown in zebrafish causes glomerular cysts and renal tubule dilation	Robinow syndrome
	Wnt6	Induces tubulogenesis, and regulates epithelial–mesenchymal interactions	‐	‐
	Wnt7b	Mediates establishment of corticomedullary axis	Wnt7b^−/−^ mice lack renal medulla	‐
	Wnt9b	Regulates mesenchymal cell differentiation and proliferation, induces MET, and regulates PCP via noncanonical pathway	Wnt9b^−/−^ mice die within 24 hours after birth, exhibit vestigial kidneys Wnt9b^‐/flox^ mice develop cystic kidneys	Bilateral renal agenesis/hypoplasia
	Wnt11	Regulates ureteric branching and nephron maturation	Forms positive feedback loop with GDNF/Ret Wnt11^−/−^ mice die within 2 days after birth, show nearly 50% reduction in glomeruli number	‐
	WLS	Mediates Wnt ligand secretion	WLS knock‐in mice exhibit cystic medullary hydronephrosis	Zaki syndrome
Membrane receptors	Frizzled (Fz)	Mediates ureteric bud growth and nephron regeneration	Fz4/Fz8 double knockout mice exhibit ureteric bud growth defects and reduced kidney size Zebrafish Fz9b mutation reduces nephron number	‐
	LRP5/6	Involved in regulation of Ret signaling network	LRP5/6 deficiency causes hypoplastic and/or cystic kidneys	‐
Cytoplasmic components	Disheveled (DVL)	Affects renal cilia development		Robinow syndrome
	GSK‐3β	Affects epithelial differentiation and nephron segregation	GSK‐3β inhibitor treatment of isolated kidney mesenchyme induces extensive epithelial differentiation	‐
	AXIN	Regulates ureteric bud/collecting duct development	‐	‐
	APC	Affects kidney development, increases renal carcinoma susceptibility	Renal epithelium‐specific Apc knockout mice develop multiple kidney cysts and renal carcinoma	‐
	β‐catenin	Regulates branching morphogenesis, ureteric formation, and nephron progenitor cell maintenance	Conditional β‐catenin deletion or overexpression causes various renal defects	‐
Nuclear components	C‐Myc	‐	‐	‐

Abbreviations: ‐, data not available; CAKUT, congenital anomalies of the kidney and urinary tract; MET, mesenchymal–epithelial transition; MM, Metanephric Mesenchyme; PCP, planar cell polarity; WD, Wolffian duct.

#### 4.1.2 PCP signaling pathway

The PCP signaling pathway, as a core branch of the noncanonical Wnt signaling pathway, plays a pivotal regulatory role in kidney development and the pathogenesis of renal ciliopathies. This pathway establishes cellular planar polarity to guide ureteric bud outgrowth and branching morphogenesis, glomerulus patterning, renal tubular alignment, and the construction of renal architecture.^104^ It works synergistically with primary cilia to mediate the cellular perception and signal transduction of mechanical stimuli, such as fluid shear stress. In the pathological process of renal ciliopathies, dysfunction of the PCP signaling pathway disrupts cilium‐dependent signal transduction, resulting in disorders of renal tubular epithelial cell polarity and alignment, thereby impairing renal filtration function and promoting cystogenesis. Furthermore, abnormal PCP signaling can activate profibrotic pathways such as the TGF‐β/Smad pathway, disturb the balance of Wnt/β‐catenin signaling, and promote renal interstitial fibrosis and cyst formation.

Inversin (INVS) acts as a pivotal switch that reprograms Wnt signaling from the canonical to the noncanonical pathway. Primary cilia, which function as mechanosensitive organelles, detect hydrodynamic forces and dynamically modulate Wnt pathway activity. Under static conditions, Wnt signaling operates predominantly through the β‐catenin‐dependent canonical pathway. In contrast, fluid shear stress triggers primary cilia‐mediated signaling events, including the upregulation of INVS expression, which promotes the degradation or sequestration of disheveled proteins. This process effectively suppresses β‐catenin‐dependent transcription and reorients signaling outputs toward noncanonical pathways, such as the PCP and Wnt/Ca^2+^ cascades^22^ (Figure 2).

### 4.2 Hedgehog signaling pathway

The Hedgehog (Hh) signaling pathway plays a critical role in embryonic kidney development, injury, ciliopathies, and the maintenance of renal tubular epithelial differentiation. Under physiological conditions, the degree of smoothened (SMO) phosphorylation determines the activation level of the Hh pathway.^105^ PTCH1 inhibits the activity of SMO. When Hh ligands bind to PTCH1, they relieve the inhibition of SMO by PTCH1. SMO is then phosphorylated by CK1 and GRK2, enters the primary cilium, and assumes an active conformation, thereby regulating downstream Glioma‐associated oncogene homolog (GLI) transcription factors to modulate the expression of various genes.^106^ Key proteins of the Hh pathway are enriched in primary cilia, and the on/off switching is critically dependent on these structures. This reliance may be attributed to the ability of primary cilia to sequester the components of the Hh cascade within the restricted luminal space, increasing their concentration by 2–3 orders of magnitude and promoting their interactions, thereby enhancing responsiveness to Hh signaling.^13^, ^107^ This process is facilitated by the cilium chamber formed by KIF7 at the cilium tip, which transports GLI1/2‐Suppressor of Fused (SUFU) to the tip for enrichment, thereby ensuring efficient signal transduction and regulation. Besides, a prior study revealed that primary cilia‐mediated Hh signaling regulates senescence in mesenchymal stem cells, diminishing osteoblastic differentiation, a process linked to changes in primary cilia length and number.^108^, ^109^

Within the Hh pathway, multiple regulatory mechanisms ensure precise control. SUFU functions as a pivotal negative regulator.^110^ In the absence of Hh signaling, SUFU binds to full‐length GLI (GLI‐FL) proteins, sequestering them in the cytoplasm to prevent their nuclear translocation and promoting their proteasomal degradation or their processing into truncated GLI transcriptional repressors.^111^ The kinesin protein KIF7 modulates GLI protein function downstream of SMO.^112^ In the absence of Hh ligands, KIF7 is phosphorylated and accumulates at the base of the primary cilium. Upon ligand binding, KIF7 is dephosphorylated and translocates with GLI to the ciliary tip, where SUFU dissociates from GLI, enabling GLI activation. The ratio of GLI activators (GLI‐A) to repressors (GLI‐R) determines Hh signaling output.^113^ GLI1 functions exclusively as an activator in a positive feedback loop. GLI2 primarily acts as an activator, whereas GLI3 predominantly acts as a repressor.^114^ In the absence of Hh ligands, SUFU facilitates GLI‐FL phosphorylation by protein kinase A (PKA), CK1, and GSK3β,^115^, ^116^, ^117^ resulting in complete degradation of GLI1 and most of GLI2, whereas GLI3 is predominantly converted to the stable repressor GLI3‐R.^118^

cAMP‐dependent PKA is a major negative regulator of Hh signaling. cAMP binds to the regulatory subunit of PKA, increasing its activity. Adenylate cyclase facilitates cAMP production, whereas phosphodiesterase promotes cAMP degradation, thereby modulating PKA activity. PKA phosphorylates GLI‐FL, thereby promoting its degradation or processing into GLI‐R. Besides, PKA phosphorylates SUFU, which stabilizes the SUFU‐GLI2/GLI3 complex. cAMP is linked to Hh signaling via the GPCR Gpr161. In the absence of Hh ligands, Gpr161 localizes to the ciliary membrane, where it activates adenylate cyclase to increase cAMP levels and suppress Hh signaling. Upon binding to the Hh ligand, Gpr161 departs from the cilium, reducing cAMP production and thereby activating the pathway.^119^

*TMEM216* functions as a central positive regulator by antagonizing SUFU, promoting GLI2‐A nuclear translocation, and preventing GLI3‐R nuclear retention while maintaining ciliary integrity and microtubule‐based transport via ARL13B and KIF3A. The TMEM216‐SUFU‐GLI axis represents a key regulatory node, offering potential therapeutic targets for ciliopathies.

**FIGURE 3:**
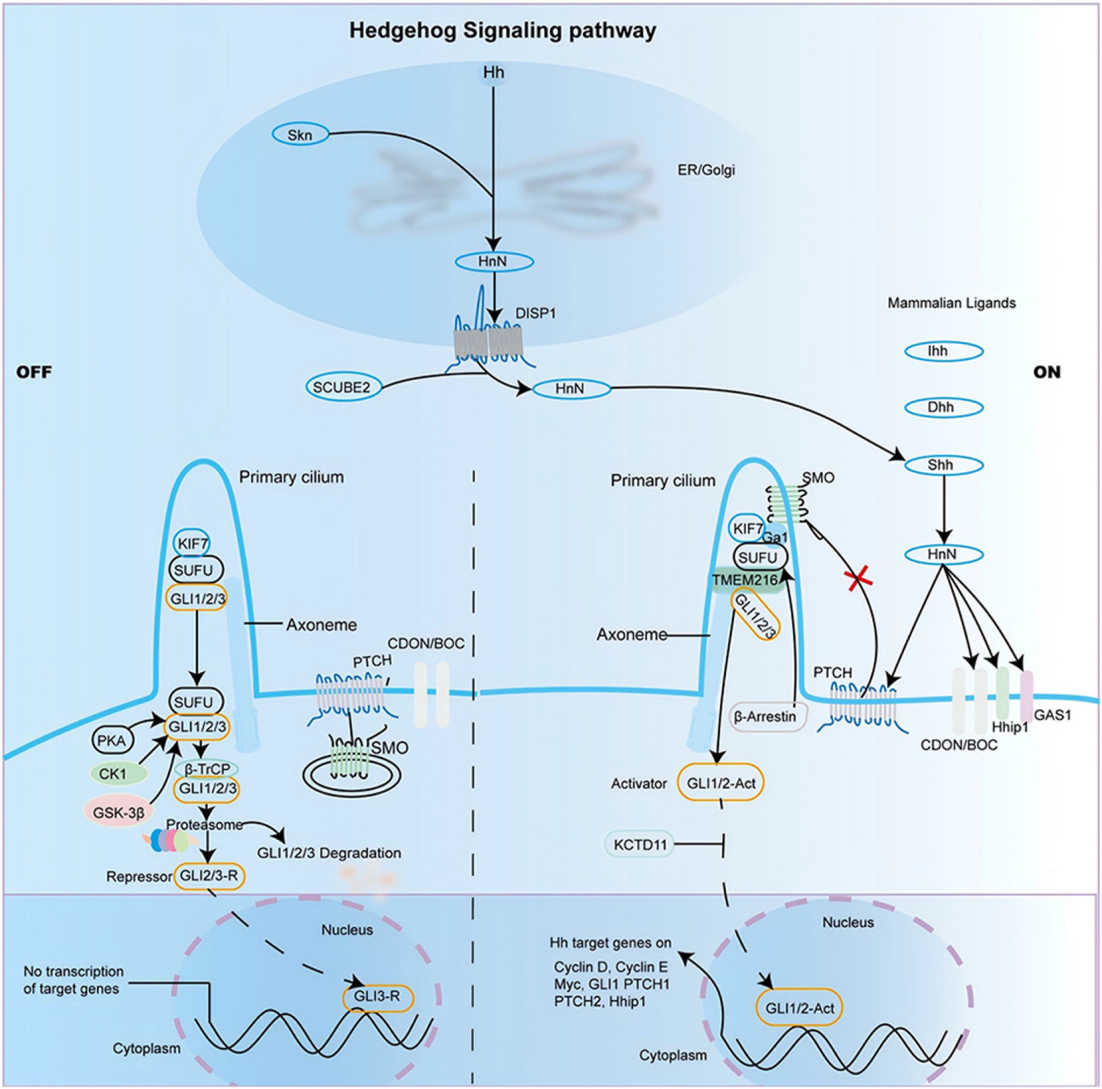
Hh signaling pathway. In the absence of Hh ligand (Off‐state), PTCH1 localizes to the cilium and represses SMO, resulting in phosphorylation of GLI transcription factors by PKA, CK1, and GSK3β. This leads to proteasome‐mediated processing of GLI into its repressor isoforms (GLI‐R), with no subsequent transcription of target genes. Upon Hh ligand binding (On‐state), PTCH1 is internalized and SMO accumulates in the cilium, promoting conversion of GLI proteins into their activator isoforms (GLI‐A). These translocate to the nucleus to drive expression of target genes, including Cyclin D, Cyclin E, Myc, GLI1, PTCH1, PTCH2, and Hhip1. Spatial regulation of Hh signaling within the primary cilium is orchestrated by core components such as PTCH1, SMO, KIF7, and the ligand‐binding complex CDON/BOC. TMEM216 functions as a key positive regulator of Hh signaling. It antagonizes SUFU, facilitates nuclear translocation of GLI2‐A, and prevents nuclear retention of GLI3‐R. GLI‐A, GLI activators; GLI‐R, GLI repressors; Hh, Hedgehog; PKA, protein kinase A.

Proper kidney morphogenesis requires a precise balance between Hh activation and repression, as evidenced by the severe reduction in nephron abundance following loss of Gli3 repressor activity.^120^ Beyond this developmental role, dysregulated Hh signaling also drives fibrosis progression in ESRD.^121^ Furthermore, Hh signaling defects are closely linked to renal ciliopathies.^122^ Mutations in NPHP‐related ciliary genes impair ciliogenesis and reduce Shh target gene expression.^123^ Consistently, animal models have confirmed that aberrant Hh signaling is a central feature in the pathogenesis of NPHP^29^ (Figure 3).

### 4.3 Notch signaling pathway

The Notch signaling pathway comprises four receptors (Notch1‐Notch4) and five canonical ligands belonging to the Delta‐like (Dll1, Dll3, and Dll4) and Jagged (Jag1, Jag2) families in mammals. Together with their downstream effectors, these components orchestrate precise gene regulatory programs in nearly all tissues of multicellular organisms. A hallmark of this process is its reliance on juxtacrine signaling, wherein ligands expressed on one cell interact with receptors on adjacent cells. This binding initiates a cascade of proteolytic cleavage culminating in the release of the Notch intracellular domain (NICD), which is translocated to the nucleus to activate the transcription of target genes.^124^, ^125^, ^126^, ^127^ It plays critical roles in embryonic development, tissue homeostasis, and disease progression. It also intersects with ciliary biology, modulating fundamental cellular functions, including proliferation, differentiation, and morphogenesis. The interaction between the Notch signaling pathway and cilium constitutes a bidirectional regulatory loop, as the primary cilium can also serve as a signaling antenna that regulates Notch pathway activity. An intact ciliary structure is correlated with upregulated Notch reporter activity and enhanced nuclear accumulation of NICD, which collectively promote normal cellular differentiation. Conversely, ciliary disruption or loss leads to attenuated Notch signal transduction and impaired differentiation.^128^ Within the kidney, it is essential for nephrogenesis and determining the fate of renin‐lineage cells.^129^ The primary cilium regulates Notch pathway activity by maintaining Ca^2+^ homeostasis. Defects in ciliary structure or function lead to decreased intracellular calcium concentrations. This, in turn, suppresses calpain activity, reducing NICD degradation. Consequently, NICD stability increases, and NICD accumulates abnormally. Sustained activation of Notch signaling then upregulates the expression of downstream target genes, driving abnormal proliferation of renal tubular epithelial cells and ultimately leading to cyst formation.

**FIGURE 4:**
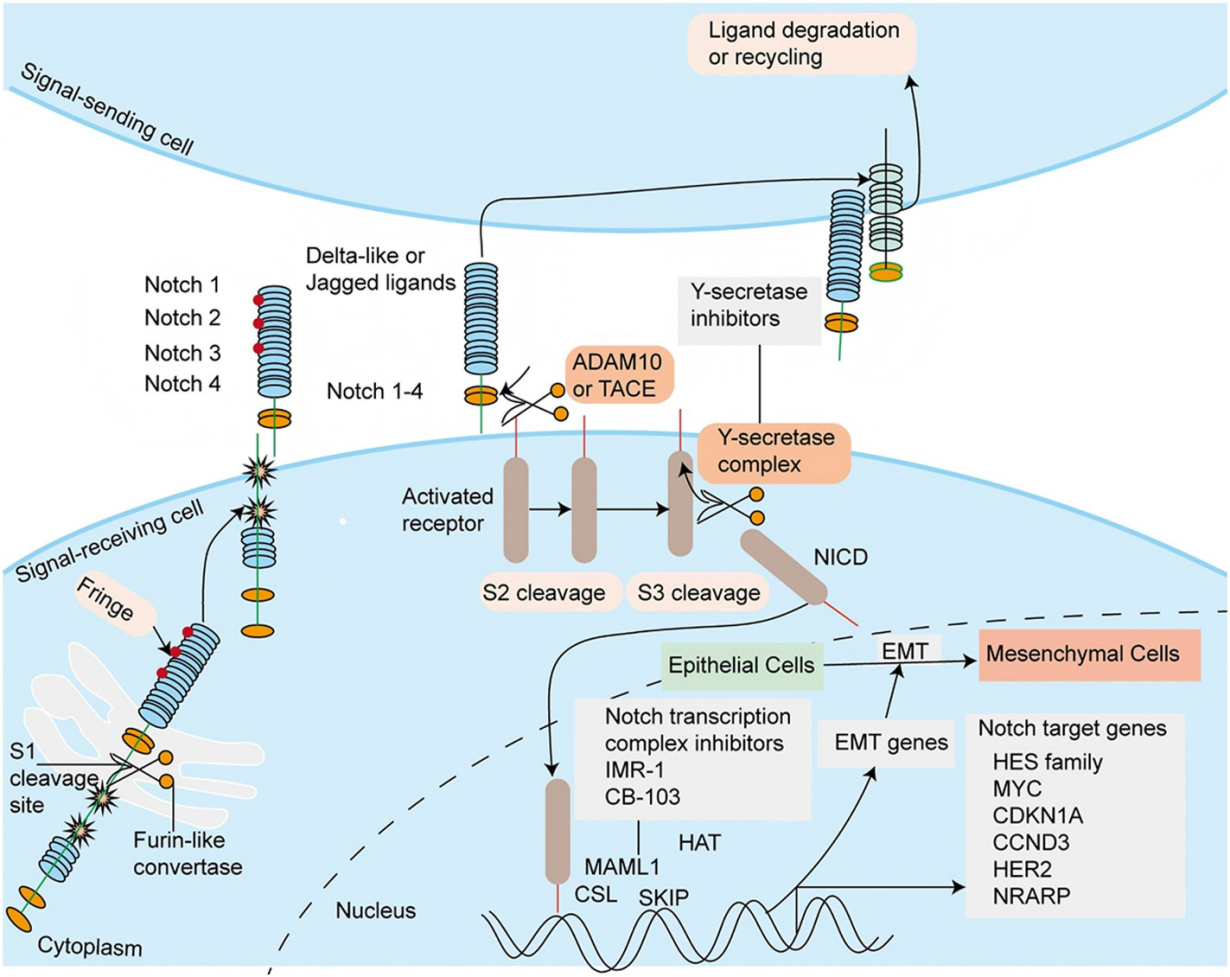
Notch signaling pathway. Notch signaling is initiated by the binding of Delta‐like or Jagged ligands on the signal‐sending cell to Notch receptors (Notch1‐4) on the receiving cell. Receptor maturation involves S1 cleavage by furin‐like convertases. Ligand binding triggers sequential proteolytic cleavages: S2 cleavage by ADAM10/TACE, followed by S3 cleavage by the γ‐secretase complex, which releases the NICD. NICD translocates to the nucleus and forms a ternary complex with CSL and MAML1, activating transcription of target genes, including HES family, MYC, CDKN1A, CCND3, HER2, and NRARP. Key inhibitors targeting this pathway include γ‐secretase inhibitors and Notch transcription complex inhibitors (e.g., IMR‐1, CB‐103). Fringe modulates ligand‐receptor binding specificity, thereby fine‐tuning pathway activity. Activated Notch signaling induces the expression of EMT‐associated genes, thereby driving the transition of epithelial cells to a mesenchymal phenotype. NICD, notch intracellular domain.

Notably, this pathway exhibits precise dose‐dependent regulatory characteristics. While excessive Notch signaling promotes cystogenesis, its loss similarly induces cyst formation by disrupting ciliary structure and function.^130^, ^131^, ^132^ These findings collectively indicate that Notch signaling must be maintained within a specific physiological activity window and that deviations in either direction disrupt renal structural homeostasis (Figure 4).

### 4.4 mTOR signaling pathway

Mammalian target of rapamycin (mTOR) signaling drives cystogenesis and progressive kidney failure in renal ciliopathies through its aberrant activation, representing a key pathogenic mechanism.^133^ Under physiological conditions, the primary cilium constrains mTORC1 activity via cilium‐localized tumor suppressors, which include LKB1, AMPK, PC1, and PC2, thereby preserving cellular quiescence.^133^ Upon disruption of ciliary integrity or function, this inhibitory control is compromised, leading to mTORC1 hyperactivation and ultimately resulting in uncontrolled proliferation of renal tubular epithelial cells, which, accompanied by metabolic dysregulation, promotes cyst formation and progressive renal enlargement.^27^

Furthermore, mTORC1 activity indirectly influences ciliary stability through autophagy regulation, establishing a self‐perpetuating cycle in which ciliary dysfunction fuels mTORC1 activation, which in turn further compromises ciliary integrity.

## 5 MODELS OF RENAL CILIOPATHIES

Renal ciliopathies represent a group of hereditary disorders arising from primary ciliary dysfunction in renal cells and are characterized by intricate pathological and genetic mechanisms. Models are of utmost importance in renal ciliopathy research.

**FIGURE 5:**
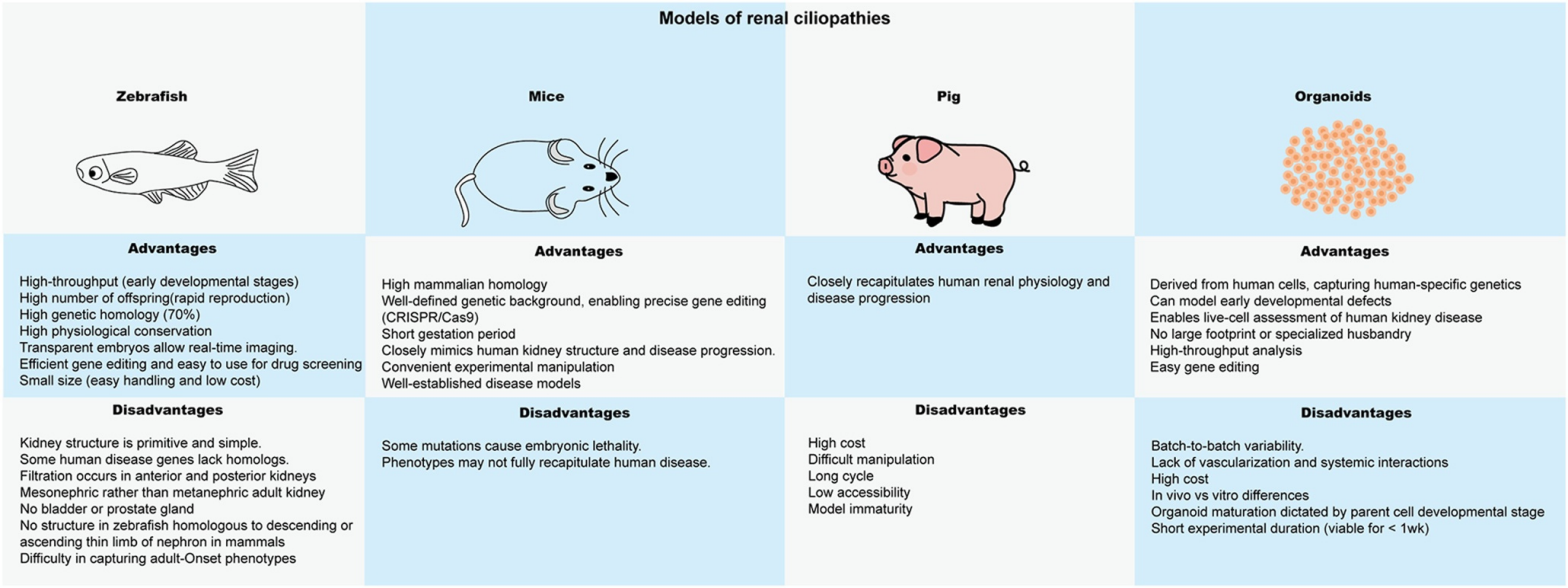
Models of renal ciliopathies. This figure illustrates the commonly used experimental models for renal ciliopathies, including zebrafish, mice, pigs, and organoids, and summarizes their advantages and disadvantages.

Given the marked differences among models in genetic homology, renal architecture, throughput, cost, tractability, and translational relevance, their main advantages and limitations are summarized in Figure 5. Among animal models, zebrafish and mice are the most widely used. Zebrafish exhibit embryonic transparency, fast breeding, rapid development,^134^ and are amenable to high‐throughput screening. However, their application is limited by the difficulty of capturing adult‐onset disease phenotypes. Mice, with high mammalian homology and well‐established disease models, are frequently utilized to generate disease models. By employing precise gene editing techniques (CRISPR/Cas9) to target knockout pathogenic genes, models that closely mimic human renal ciliopathy phenotypes can be created.^135^ However, certain mutations are embryonically lethal, and the observed phenotypes do not always fully represent the full spectrum of the human disease. Compared to rodents, pigs more closely recapitulate human renal physiology and disease progression, but are costly, challenging to manipulate, less accessible, and remain immature as a model.^136^ Organoids, derived from patient‐specific or gene‐edited pluripotent stem cells, form nephron like three‐dimensional structures^137^ and enable high‐throughput screening and personalized mechanistic studies, excelling at simulating human kidney structure and function.^138^ However, they lack vascularization, fluid flow, and a renal microenvironment, representing an immature developmental stage. Collectively, no single model recapitulates the full spectrum of human renal ciliopathies. Each has distinct strengths and limitations, and the optimal choice depends on the research question.

## 6 THERAPEUTIC STRATEGIES FOR TREATING RENAL CILIOPATHIES

The identification of an expanding repertoire of genes implicated in renal ciliopathies, coupled with advances in prenatal diagnostic methods, has significantly improved our understanding of this complex disease spectrum. They present with diverse clinical manifestations, ranging from renal cystogenesis to progressive renal failure, posing a substantial threat to patient survival and quality of life. Leveraging breakthroughs in genetic research and the precision afforded by prenatal diagnostics, we can now perform earlier disease risk stratification, providing patients with invaluable therapeutic windows. Conventional therapeutic approaches have historically relied on symptomatic management and organ support modalities, which primarily serve to mitigate ESRD progression and relieve symptoms. However, these interventions exhibit limited efficacy in arresting the cascading pathogenic mechanisms underlying ciliopathy progression. The exploration and clinical translation of innovative therapeutic strategies represent an urgent clinical imperative. These cilia‐targeted treatments hold the transformative potential to overcome the limitations of traditional approaches, propelling disease management paradigms beyond mere attenuation of disease progression toward the ambitious goal of functional tissue restoration and regenerative medicine applications.

Therapeutic paradigms for renal ciliopathies have undergone transformative exploration, integrating multidisciplinary advances to address pathogenic mechanisms across genetic, epigenetic, and signaling axes. At the forefront, precision genome editing employs CRISPR‐Cas9 systems conjugated with kidney‐specific ligands, allowing targeted correction of pathogenic mutations.^139^ This tissue‐specific genetic editing has the potential to selectively mitigate ciliary pathology in ciliopathies by precisely restoring ciliary structural integrity and functional competence. Complementary epigenetic strategies utilize HDAC6 inhibitors^140^, ^141^, ^142^ to stabilize microtubule dynamics and rescue ciliary architecture and IFT function, which is a critical intervention given the role of ciliary disassembly in disease progression.

Modulation of signal transduction can further enhance therapeutic efficacy through synergistic combination regimens. In animal models, dual blockade of hyperactivated cAMP‐PKA‐mTOR pathways with mTOR inhibitor and vasopressin V2 receptor antagonists^143^ suppressed cyst growth and preserved renal function by inhibiting ERK phosphorylation and c‐Myc activation. In Ttc21b‐mutant models, purmorphamine activates Hh signaling to rescue defects in CEP290 mutants, whereas Gant61 antagonizes Hh signaling to reduce cyst formation. Besides, cell cycle inhibition via roscovitine and Yes‐associated protein (YAP) inhibition with verteporfin, have demonstrated efficacy in preclinical NPHP models.^27^ In renal fibrosis, the Wnt/β‐catenin pathway has emerged as a central therapeutic target. Endogenous and small‐molecule inhibitors of key pathway nodes and natural products from traditional Chinese medicine suppress Wnt/β‐catenin signaling for antifibrotic effects.^144^ Pharmacological inhibition of mTOR with rapamycin has been demonstrated to suppress cyst growth and delay renal function decline.^145^

Besides, modulators of transmembrane channels, inflammation, and cellular metabolism have shown potential as therapeutic targets for renal ciliopathies, while AVPR2 antagonists are the only approved approach. Magnetically guided iron oxide nanoparticles (Fe_2_O_3_‐NPs),^146^ coupled with mechanosensitive channel modulation, represent a critical advancement in drug delivery, doubling therapeutic efficacy. In vitro studies have demonstrated that 4‐phenylbutyric acid (4‐PBA) may act as a molecular chaperone to rescue mutant WLS expression, whereas small‐molecule Wnt agonists can partially reverse developmental defects.^84^ In the field of regenerative medicine, animal models have demonstrated that the transplantation of mesenchymal stem cells overexpressing Wnt‐5a enhances bladder tissue regeneration.^147^

**FIGURE 6:**
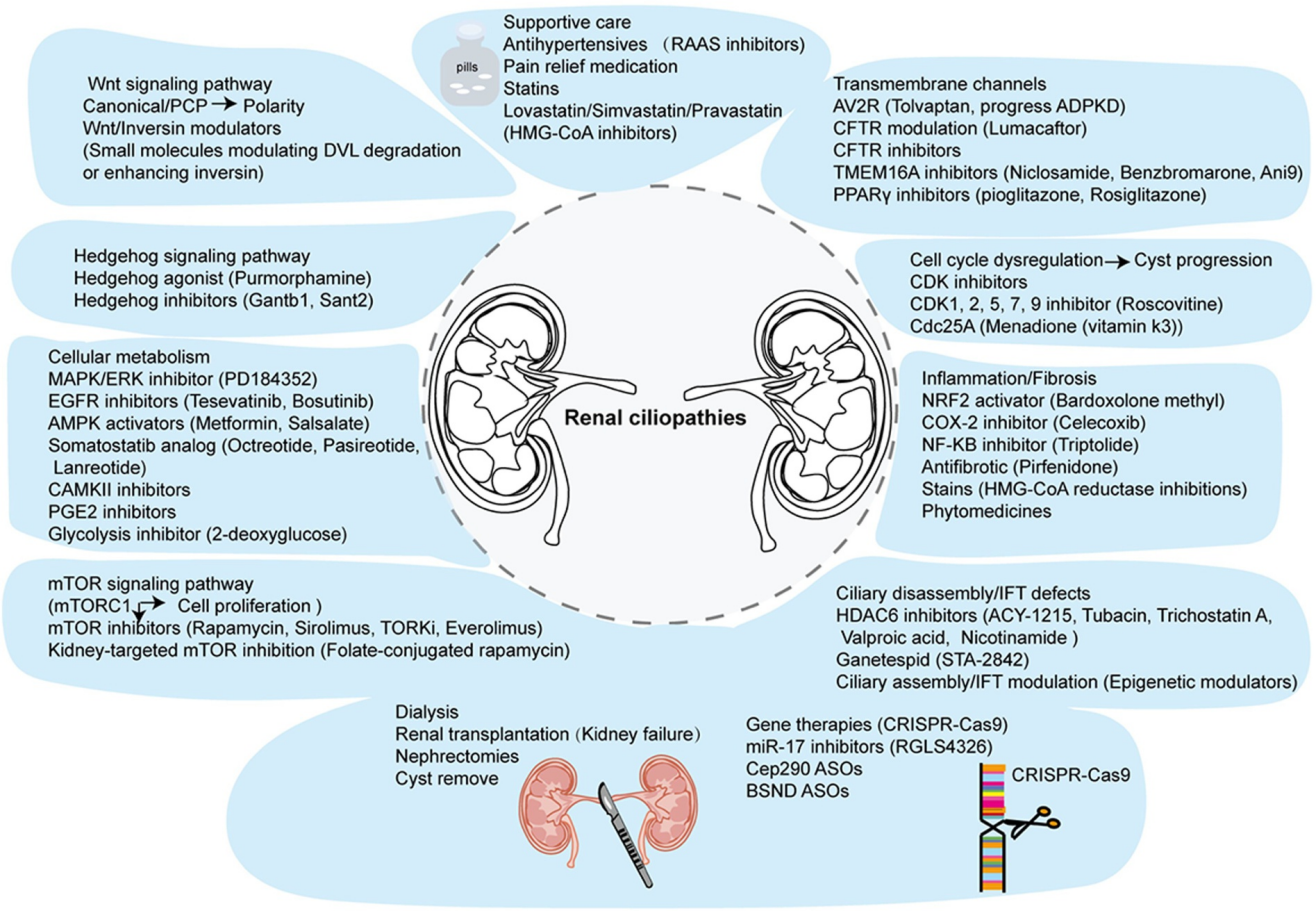
Emerging therapeutic strategies and potential therapeutic targets in renal ciliopathies. This figure illustrates selected therapeutic strategies currently under investigation for renal ciliopathies. The central circle represents renal ciliopathies, surrounded by functional modules corresponding to key signaling pathways and pathological processes that have been implicated in disease pathogenesis: mTOR signaling, Wnt signaling, Hedgehog signaling, cellular metabolism, ciliary disassembly/IFT defects, inflammation/fibrosis, cell cycle regulation, transmembrane channels, surgeries, and gene therapies. Each module is linked to representative therapeutic approaches. Many of these strategies remain confined to evaluation in animal models or early‐stage clinical trials. IFT, intraflagellar transport; mTOR, mammalian target of rapamycin.

This multimodal therapeutic framework spans genome editing, epigenetic remodeling, signal transduction blockade, molecular chaperones, regenerative medicine, and precision nanotechnology, which underpins the transition from symptomatic management to mechanism‐based curative interventions (Figure 6).

## 7 CONCLUSION AND PERSPECTIVE

This review systematically examines ciliary structure and function, the spectrum of renal ciliopathies, and the underlying signaling pathways. We further evaluate current disease models, emerging therapeutic strategies, and potential therapeutic targets. Proper ciliary architecture is essential, as it allows the organelle to function dually as molecular motor and signaling hub; structure compromise can trigger cascading functional collapse. Cilia transduce extracellular signals into intracellular signaling cascades via specialized receptors, modulating pathways such as Wnt, Hedgehog, Notch, and mTOR signaling. These pathways form a complex regulatory network that coordinates tubular differentiation, cell cycle arrest, and PCP. Ultimately, aberrations in ciliary structure, function, or signaling integration drive the pathogenesis of renal ciliopathies. However, the intricate signaling networks disrupted by ciliary dysfunction remain poorly understood in terms of their synergistic or antagonistic interactions. Existing studies have primarily focused on unidirectional modulation of individual pathways. With advances in molecular biology, we can investigate the molecular mechanisms underlying renal ciliopathies using in vivo and in vitro models. On this basis, systematically elucidating the molecular mechanisms and aberrant signaling pathways underlying renal ciliopathies can help us better understand their pathogenesis.

Finally, we summarize the emerging targets and therapeutic strategies derived from functional modules linked to key signaling pathways and pathological processes implicated in disease pathogenesis. Although most of them remain confined to animal models or preclinical trials, they provide vital insights for developing novel therapies.

Future research must prioritize multiomics integration to delineate the developmental trajectories of ciliated renal epithelial cells through single‐cell transcriptomic, epigenomic, and spatial profiling, thereby identifying critical time windows and regulatory hubs in cystogenesis. Looking ahead, machine‐learning‐driven analyses of protein interaction networks hold promise for pinpointing novel pathogenic genes based on topological features. Clinically, we can improve prognostic accuracy by expanding multicenter cohorts, standardizing renal anomaly classifications, and integrating longitudinal data. Parallel efforts should accelerate the discovery of novel biomarkers for early risk stratification, disease prediction, diagnosis, and prevention. With advances in artificial intelligence (AI), integrating ultrasonography with AI holds promise for improving detection accuracy and efficiency in prenatal diagnosis of renal ciliopathies. Deep learning algorithms, trained on large‐scale ultrasound image datasets, can help detect subtle renal structural anomalies, potentially reducing operator‐dependent errors. Furthermore, AI may extract intricate morphological details from ultrasound images, enabling earlier detection of renal pathologies during gestation.

In summary, elucidating the etiology and signaling networks of renal ciliopathies, identifying feasible and effective therapeutic targets, and ultimately achieving a therapeutic shift from “slowing disease progression” to “restoring ciliary function” constitute the goals of future research. Although challenges remain, the ongoing convergence and application of multiomics technologies, gene‐editing technologies, organoid models, and AI hold promise that this goal will be gradually attained.

## AUTHOR CONTRIBUTIONS

All authors contributed to the article and approved the submitted version. Xiaofan Zhu, Qiaowei Zhang, Xiangdong Kong and Qi Feng conceptualized and wrote the manuscript. Xiaofan Zhu, Qiaowei Zhang, Qi Feng, Shuwen Xue, Zhi Gao, Panlai Shi, Li Wang, and Xiangdong Kong reviewed and revised the manuscript. Xiaofan Zhu, Qi Feng and Xiangdong Kong made substantial contributions to the design and critical revision of the manuscript.

## CONFLICT OF INTEREST STATEMENT

The authors declare no conflicts of interest.

## ETHICS STATEMENT

Not applicable.

## Data Availability

The data used to support the findings of this study are included within the paper.
